# A Case of B-Cell Lymphoblastic Lymphoma Presenting with an Isolated Epidural Mass Treated Successfully with Radiotherapy Followed by United Kingdom Acute Lymphoblastic Leukemia (UKALL) Chemotherapy Protocol

**DOI:** 10.3390/hematolrep16040069

**Published:** 2024-11-23

**Authors:** Musa Fares Alzahrani

**Affiliations:** 1 Department of Medicine, College of Medicine, King Saud University, Riyadh 12372, Saudi Arabia; malzahrani@ksu.edu.sa; Tel.: +966-566041113; 2 Oncology Center, King Saud University Medical City, Riyadh 11451, Saudi Arabia

**Keywords:** lymphoblastic lymphoma, UKALL, LBL, B-LBL, epidural

## Abstract

Background: B-cell lymphoblastic lymphoma (B-LBL) is an aggressive type of non-Hodgkin lymphoma that usually involves lymph nodes, skin and soft tissue. Bone marrow and peripheral blood are normally spared from involvement in the disease. B-LBL typically forms solid masses that have similar pathologic and immunophenotypic features to their liquid counterpart, B-cell acute lymphoblastic leukemia (B-ALL). The presentation of B-LBL with a solitary epidural mass at the cervical spine is very rare and the optimal treatment of such cases is unknown. Most of the literature on the management of B-LBL comes from small case series, pediatric patients, or as part of retrospective data that combine B-LBL with B-ALL cases. Case presentation: The case presented herein is a unique presentation that was treated using three modalities, namely surgical resection, radiotherapy and consolidation with systemic chemotherapy, adopted from the United Kingdom acute lymphoblastic leukemia (UKALL14) protocol. Conclusions: The patient attained complete remission following the planned treatment and is still in remission for more than four and half years from the time of his initial diagnosis.

## 1. Introduction

B-lymphoblastic leukemia (B-ALL)/lymphoma (B-LBL) is recognized in the 5th edition of the World Health Organization (WHO) classification of hematolymphoid tumors as one of the precursor B-cell neoplasms [1]. When the disease presents primarily with nodal or extranodal involvement, the term lymphoma (B-LBL) is used; however, if there is extensive bone marrow and peripheral blood involvement, the term used is leukemia (B-ALL). Most cases of B-ALL/LBL present with leukemia and only 10–20% of cases present as lymphoma [2]. Lymphoblastic lymphoma (LBL) of T-cell origin (T-LBL) is more common than B-LBL, which only constitutes about 10% of all LBLs [3]. Unlike T-LBL, which typically presents at an advanced stage at the time of diagnosis, B-LBL often presents with localized disease [4]. The usual presentation of B-LBL includes the involvement of lymph nodes, skin, soft tissue, bone and, rarely, the mediastinum [3,5]. Solitary epidural involvement is rare. Histologically and immunophenotypically, these two entities, namely B-ALL and B-LBL, are essentially identical, although subtle differences can be seen at the molecular genetic level [6]. Typically, the neoplastic cells are composed of small- to medium-size immature blast cells with scant cytoplasm and moderately condensed chromatin. Immunophenotype studies, such as those that perform flowcytometry and/or immunohistochemistry analyses of B-LBLs, confirm their B-cell origins by observing the positive expression of B-cell markers, such as CD19, cCD79a and cCD22, in addition to the expression of the immature marker TdT. Since B-LBL cases are rare, they are typically treated in a similar way to B-ALL. The prognosis of B-LBL in adults is not entirely clear, but it is largely extrapolated from B-ALL or the pediatric literature. It is believed that the prognosis of B-LBL is relatively favorable and becomes less favorable as patients become older [7,8]. The optimal treatment of patients with B-LBL who present with solitary lesions, especially those presenting as an epidural mass, is unclear.

Whether surgical removal or localized radiation is sufficient remains unclear. It is usually preferred to treat these cases with systemic chemotherapy, with the idea of killing any possible microscopic disease that could potentially disseminate to other tissues if left untreated. Given how rare B-LBL is in adults and the limited information available regarding optimal treatment and prognosis, the reporting of such cases is important to enrich our knowledge.

Here, a report of a unique and rare presentation of a B-LBL case is presented in detail. The case was treated surgically, after which radiation and systemic treatment were initiated, with an intensive chemotherapy protocol adopted from the UKALL14 protocol [9]. The patient attained a complete remission (CR) and is currently alive, having remained in a stable condition for more than four and half years.

## 2. Methods

This is a case report that used retrospective data accessed through the hospital electronic medical records. Details of the patient’s clinical, radiological and pathological features were collected. Informed consent was given by the patient and the study was approved by the institutional review board (IRB) on October 6th 2024, with a reference number 24/1605/IRB.

## 3. Case Description

**Figure 1:**
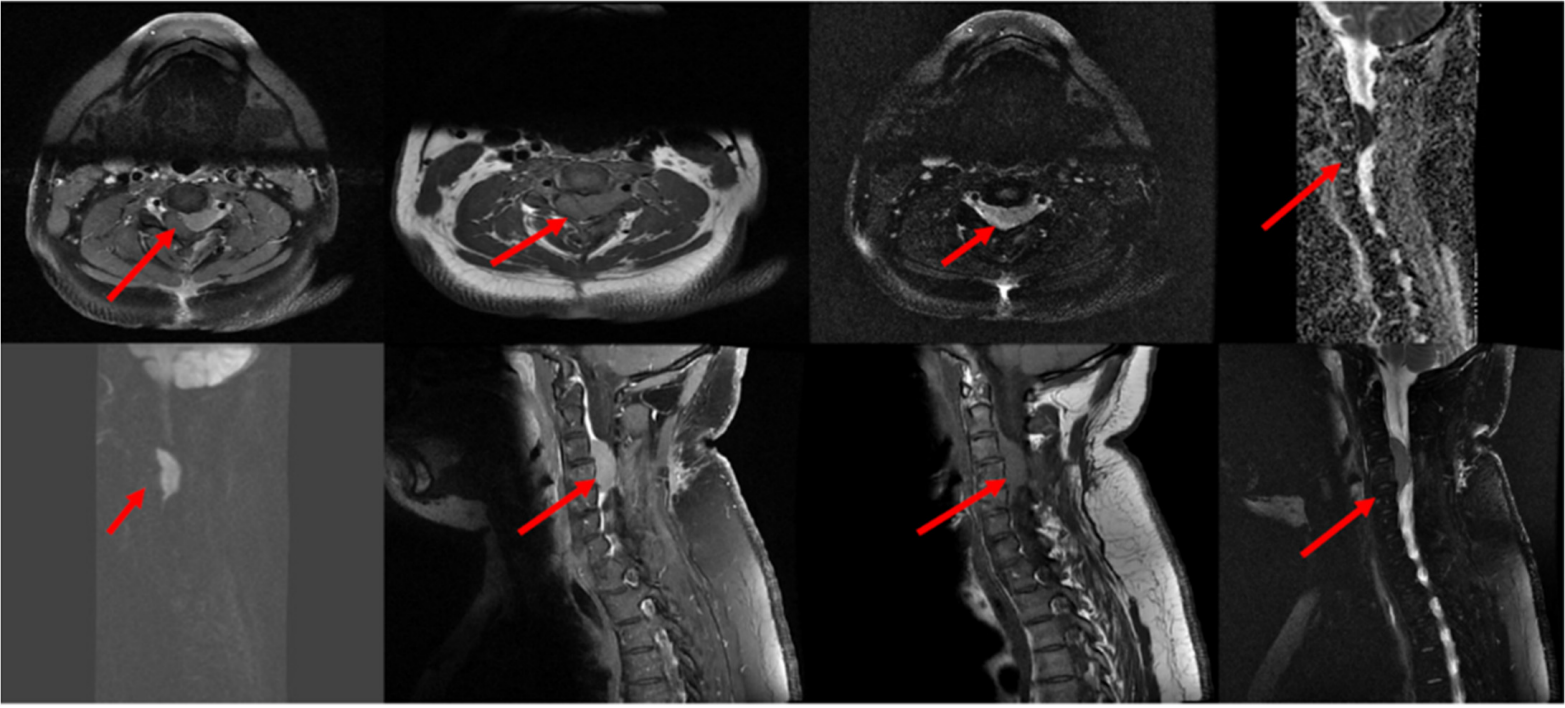
MRI of the cervical spine showed that there is an intraspinal epidural mass (red arrows) measuring 1.2 × 1.8 × 3.2 mm (AP, TV and CC diameters) in the ventrolateral aspect at the level of C3–C4 extending to the left neural foramen, impinging the adjacent nerve root and compressing the spinal cord with cervical myelopathy. The mass demonstrates avid homogeneous enhancement in postcontrast study and DWI restriction. Abbreviation: Magnetic resonance imaging (MRI), anteroposterior (AP), transverse (TV), and craniocaudal (CC) planes.

The patient is a 30-year-old Saudi male who was previously healthy without any past comorbidities or family history. He presented in December 2019 with a three-month history of neck pain and stiffness, associated with changes in his handwriting, upper limb numbness and a difficulty with fine motor skills. He presented to another local hospital, and their investigations included a complete blood count (CBC) that revealed a hemoglobin of 151 g/L, mean corpuscular volume of 91 fL (normal: 80–94), platelets of 342.0 × 10^9^/L, a total white blood cell count (WBC) of 8.1 × 10^9^/L and an absolute neutrophil count (ANC) of 5.9 × 10^9^/L. The coagulation profile showed a normal international normalized ratio (INR) of 0.96, a prothrombin time (PT) of 13.5 s and a mildly elevated partial thromboplastin time (PTT) of 36 (normal: 25–35) s. A peripheral blood film confirmed the absence of abnormal cells or blasts. His level of alanine aminotransferase was 33 units/L, aspartate transaminase was 14 units/L, albumin was 34.5 g/L, total bilirubin was 4.4 μmol/L, alkaline phosphatase was 85 unit/L, gamma-glutamyltransferase was 15 unit/L, lactate dehydrogenase was 298 unit/L (normal 87–241), sodium was 145 mmol/L, chloride was 106 mmol/L, potassium was 4 mmol/L, phosphorus was 1.03 mmol/L, corrected calcium was 2.3 mmol/L, creatinine was 73 micromoles/L and his level of blood urea nitrogen was 3.4 mmol/L. A qualitative *BCR-ABL1* gene test was performed using peripheral blood, based on a polymerase chain reaction test, and came back negative. Magnetic resonance imaging (MRI) was performed and showed an enhancing soft tissue mass within the anterior epidural space at the level of cervical vertebral body (C) C3–C4, with the possibility of impingement of the adjacent nerve root on the left side (see Figure 1).

**Figure 2:**
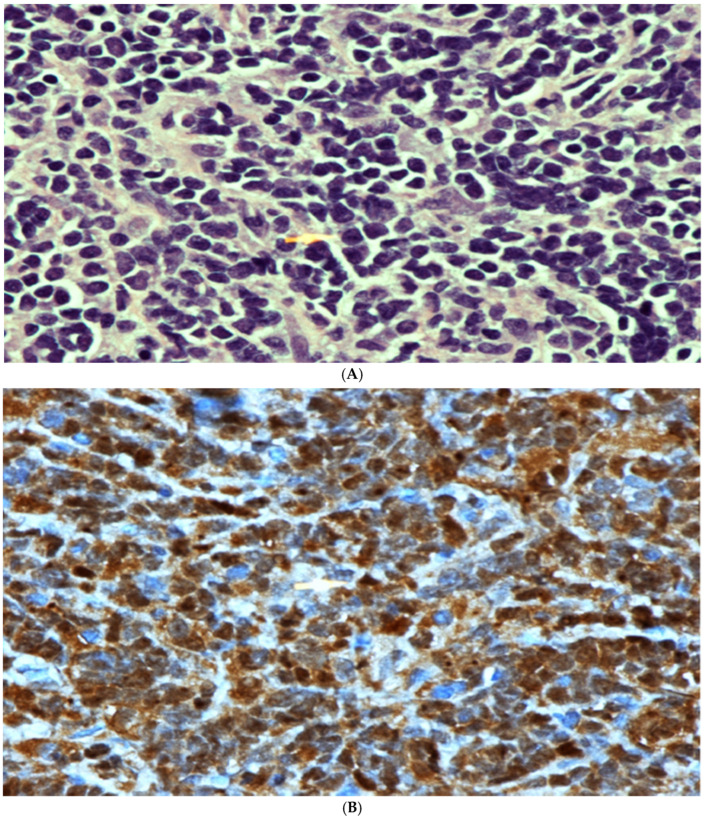

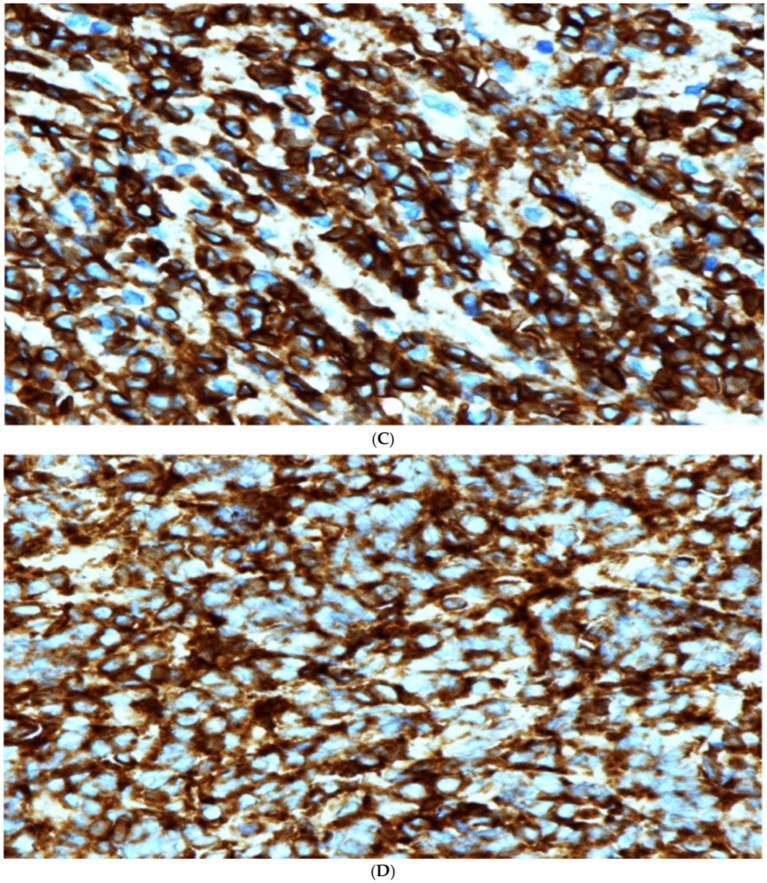
(**A**) High power microscopic view of lymphoblastic lymphoma. Note the presence of numerous monotonous lymphoid cells showing irregular nuclei, fine nuclear chromatin and conspicuous nucleoli in some cells. [Hematoxylin/Eosin (H/E) stain ×600 magnification (Mag)]. (**B**) TdT Immunohistochemical stain. Note the strong positive nuclear staining. Mag ×600. (**C**) Strong positive membranous staining of the lymphoma cells with CD79A confirming the B cell lineage of these cells. Mag ×600. (**D**) Strong positive cytoplasmatic and membranous staining with CD99 stain. This stain, although not specific, is commonly positive in cases of lymphoblastic lymphoma. Mag ×600.

At the beginning of February 2020, the patient underwent anterior decompression and lesion resection at C3–C4 and a laminectomy at C5. A large panel of immunohistochemical stains were performed on the pathologic sample and it showed that the tumor cells were strongly positive for TdT, CD99, CD79a, PAX5, CD43, CD10, CD45, FLI1, CD20, CD3 and CD34. The stains for synaptophysin, NSE, WT1, myogenin, MPO, CD2, CD5, CD8 and CD1a are all negative. The proliferation marker Ki67 was positive in 70% of tumor cells, while the PAS stain was negative (see Figure 2).

Bone marrow aspiration and biopsy were also performed and showed a normocellular marrow with active trilineage hematopoiesis, with no morphological or immunophenotypical evidence of an increase in blasts or dysplasia.

In addition to the aforementioned MRI, a computed tomography scan (CT) of the neck/chest/abdomen and pelvis was performed and showed no evidence of disease elsewhere, as well as the absence of any significant lymphadenopathy. Positron emission tomography (PET) was also performed and showed no other areas of any suspicious uptake.

After his surgery, the patient was referred for radiotherapy and was prescribed 2500 centi-gray (cGy) in five fractions to the C spine.

The patient was then started on the United Kingdom acute lymphoblastic leukemia 14 protocol (UKALL14) in June 2020 [9]. Phase I induction (28 days) comprised weekly rituximab at 375 mg/m^2^, in addition to weekly daunorubicin at 30 mg/m^2^ and vincristine at 1.4 mg/m^2^, dexamethasone (four days on and four days off) at a dose of 10 mg/m^2^, peg-asparaginase at 1000 IU/m^2^ on days 4 and 18 and intra-thecal methotrexate at 12.5 mg once on day 14. On 8 July 2020, a follow-up MRI of the cervical spine was carried out after the completion of induction chemotherapy, which showed a significant improvement in and resolution of the anterior intraspinal epidural mass at the level of the C3 and C4 vertebral bodies. The patient then completed the rest of the protocol in a similar way to that detailed fully in the UKALL14 publication [10]. The protocol contained the following phases: phase I and II inductions, intensification/central nervous system (CNS) prophylaxis with high-dose methotrexate, consolidation cycles 1–2 and 4, a delayed intensification phase and maintenance therapy for 2 full years.

**Figure 3:**
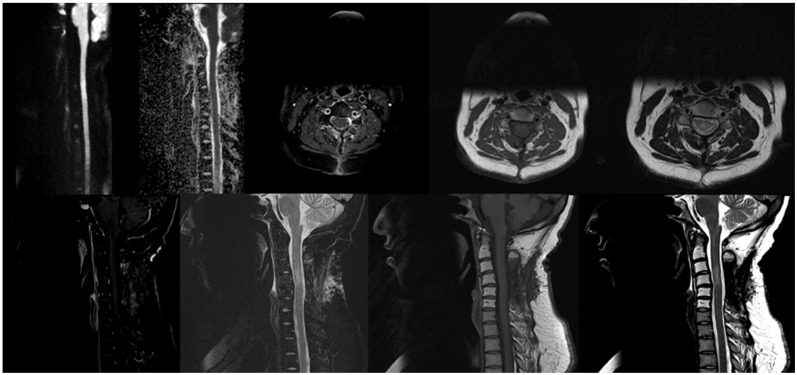
Follow up MRI Showed that there has been interval laminectomy performed at C3–C5 levels, resolution of the large intraspinal anterior epidural mass on the ventrolateral aspect of the canal at the level of C3–C4, improvement of the signal alteration within spinal cord due to cord myelopathy, no significant diffusion restriction, stable faint mild enhancement of C4 vertebra and new alteration on T1 and T2-weighted images at C2 to C6 vertebrae related to radiotherapy.

After the completion of the patient’s chemotherapy protocol, a repeat MRI was carried out on 15 May 2023 and showed the persistent complete resolution of the C3–C4 anterior epidural mass without evidence of recurrence (see Figure 3).

## 4. Discussion

B-LBL is a very aggressive type of B-cell lymphoma, which typically presents rapidly and involves the lymph nodes, skin and/or other soft tissues. Other possible, albeit rare, sites of involvement include liver, renal, testicular, rectal or small bowel [11,12,13,14,15].

Epidural involvement in the primary site of the disease is extremely rare. There are few cases reported in the literature with such a presentation. In one of the rare studies found in the literature, Nambier et al. reported a case wherein paraparesis secondary to a spinal lesion was later diagnosed as B-LBL; however, the treatment of that case was different from ours [16]. In that study, the patient was treated with combination chemotherapy, using the cyclophosphamide, vincristine, adriamycin and dexamethasone (hyper-CVAD) protocol.

Unfortunately, the natural history, prognosis and optimal treatment of B-LBL in adults is unknown and largely derived from case reports and retrospective series or extrapolated from the pediatric literature on B-ALL/LBL [17]. Nevertheless, multi-agent chemotherapy regimens are usually used in a similar fashion to B-ALL [18]. One important difference, however, lies in the inability to measure MRD accurately in B-LBL, which makes the transplantation decision difficult. The documentation of complete remission through different means such as imaging modalities, namely MRI in our case, is therefore of paramount importance [19]. Presumably, positron emission tomography (PET) scans can also be used to assess treatment responses, although there is not enough evidence to support this practice [20,21]. It is also unknown whether surgery and/or radiation alone is sufficient in truly localized disease. It is assumed that systemic treatment is still needed to clear microscopic disease, similar to the treatment of other limited-stage aggressive B-cell lymphomas, such as Burkitt or diffuse large B-cell lymphoma.

Several pediatric studies have included patients with B-LBL, but the numbers included were very small. Regimens used for pediatric B-LBL patients typically comprise the LSA2L2 (cyclophosphamide, vincristine, methotrexate, prednisone, daunomycin, cytarabine, thioguanine, asparaginase, hydroxyurea and carmustine) protocol or a modification of it. For instance, the Children’s Oncology Group (COG) protocol A5971, using a modified LSA2L2 regimen, reported the outcomes of 60 patients with B-LBL, the majority of which presented with localized disease and reported an excellent 5-year overall survival (OS) rate of 96%, with an event-free survival (EFS) rate of 90% [22].

The use of the UKALL14 protocol in adults has been shown to be effective in B-ALL [9]. We opted to treat our patient with this protocol, which, at the time, was our standard ALL protocol for adult patients.

The patient tolerated treatment reasonably well. He achieved complete remission, remained stable and is likely cured from the disease. His last follow-up was more than 4.5 years after his diagnosis.

The current case report helps enrich the literature by providing some guidance on how to manage such rare cases. Despite the importance of this case, some limitations exist. This includes a lack of full genetic tests, which were not performed since the bone marrow was not involved and since the access to further tests was limited at the time of the patient’s diagnosis.

## 5. Conclusions and Future Directions

The epidural presentation of B-LBL is rare. Its treatment should involve a multidisciplinary team that includes neurosurgery, radiation oncology and hematology. Surgical decompression with or without radiation is frequently needed to rapidly reverse compression symptoms, followed by systemic multi-agent chemotherapy to cure the underlying microscopic disease. It remains unknown whether this unique presentation of epidural mass is associated with specific risk factors or genetic aberrations; future studies could help answer this question.

## Data Availability

The data presented in this study are available on request from the corresponding author due to privacy.
